# 3,3′-Di-*n*-propyl-1,1′-(1,3-phenyl­ene­dimethyl­ene)di(1*H*-imidazol-3-ium) bis­(hexa­fluorophosphate)

**DOI:** 10.1107/S1600536812026955

**Published:** 2012-06-20

**Authors:** Rosenani A. Haque, S. Fatimah Nasri, Mohd Mustaqim Rosli, Hoong-Kun Fun

**Affiliations:** a School of Chemical Sciences, Universiti Sains Malaysia, 11800 USM, Penang, Malaysia; b X-ray Crystallography Unit, School of Physics, Universiti Sains Malaysia, 11800 USM, Penang, Malaysia

## Abstract

In the title compound, C_20_H_28_N_4_^2+^·2PF_6_^−^, the dihedral angles between the benzene ring and the imidazole rings are 70.18 (11) and 69.83 (11)°, while the imidazole rings form a dihedral angle of 40.52 (12)°. In the crystal, weak C—H⋯F inter­actions link the mol­ecules into a two-dimensional network parallel to (001). A π–π inter­action with a centroid–centroid distance of 3.601 (1) Å is also observed in the crystal structure.

## Related literature

For related structures, see: Haque *et al.* (2010 ▶, 2011 ▶).
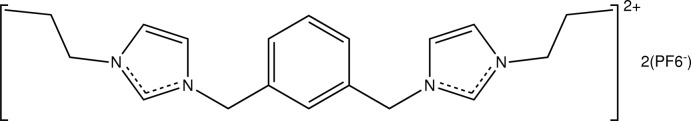


## Experimental

### 

#### Crystal data

C_20_H_28_N_4_^2+^·2PF_6_^−^
*M* *_r_* = 614.40
Triclinic, 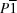

*a* = 7.2623 (2) Å
*b* = 11.3926 (3) Å
*c* = 15.9191 (4) Å
α = 86.157 (1)°
β = 80.917 (2)°
γ = 88.946 (2)°
*V* = 1297.61 (6) Å^3^
*Z* = 2
Mo *K*α radiation
μ = 0.27 mm^−1^
*T* = 100 K
0.33 × 0.15 × 0.07 mm

#### Data collection

Bruker SMART APEXII CCD area-detector diffractometer
Absorption correction: multi-scan (*SADABS*; Bruker, 2009 ▶) *T*_min_ = 0.916, *T*_max_ = 0.980
20758 measured reflections
7499 independent reflections
5364 reflections with *I* > 2σ(*I*)
*R*_int_ = 0.037

#### Refinement

*R*[*F* ^2^ > 2σ(*F* ^2^)] = 0.051
*wR*(*F* ^2^) = 0.126
*S* = 1.04
7499 reflections
345 parameters
H-atom parameters constrained
Δρ_max_ = 0.60 e Å^−3^
Δρ_min_ = −0.47 e Å^−3^

### 

Data collection: *APEX2* (Bruker, 2009 ▶); cell refinement: *SAINT* (Bruker, 2009 ▶); data reduction: *SAINT*; program(s) used to solve structure: *SHELXTL* (Sheldrick, 2008 ▶); program(s) used to refine structure: *SHELXTL*; molecular graphics: *SHELXTL*; software used to prepare material for publication: *SHELXTL* and *PLATON* (Spek, 2009 ▶).

## Supplementary Material

Crystal structure: contains datablock(s) I, global. DOI: 10.1107/S1600536812026955/lh5488sup1.cif

Structure factors: contains datablock(s) I. DOI: 10.1107/S1600536812026955/lh5488Isup2.hkl

Additional supplementary materials:  crystallographic information; 3D view; checkCIF report

## Figures and Tables

**Table 1:** Hydrogen-bond geometry (Å, °)

*D*—H⋯*A*	*D*—H	H⋯*A*	*D*⋯*A*	*D*—H⋯*A*
C1—H1*A*⋯F3^i^	0.95	2.32	3.238 (3)	162
C2—H2*A*⋯F9^ii^	0.95	2.46	3.274 (3)	144
C3—H3*A*⋯F10^iii^	0.95	2.45	3.291 (2)	148
C3—H3*A*⋯F11^iii^	0.95	2.49	3.344 (2)	150
C4—H4*B*⋯F9^i^	0.99	2.51	3.195 (2)	126
C10—H10*A*⋯F11^iii^	0.95	2.54	3.405 (2)	151
C18—H18*B*⋯F7^iv^	0.99	2.43	3.324 (3)	149

Symmetry codes: (i) 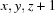; (ii) 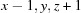; (iii) 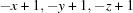; (iv) 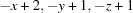.

**Fig. 1.**
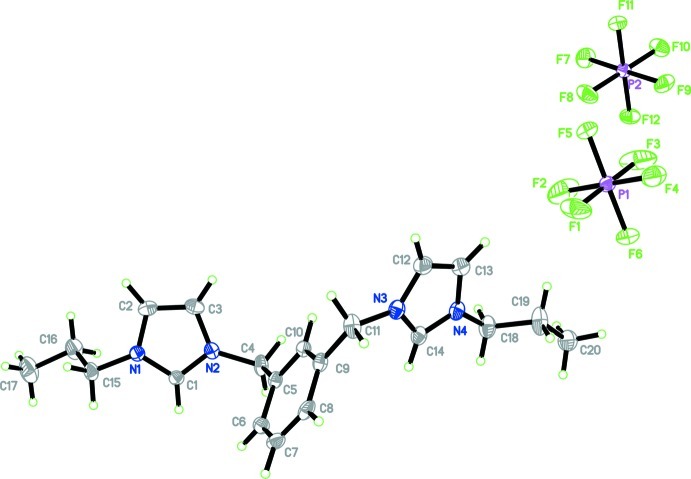
The molecular structure, showing 50% probability displacement ellipsoids. Hydrogen atoms are shown as spheres of arbitrary radius.

**Fig. 2.**
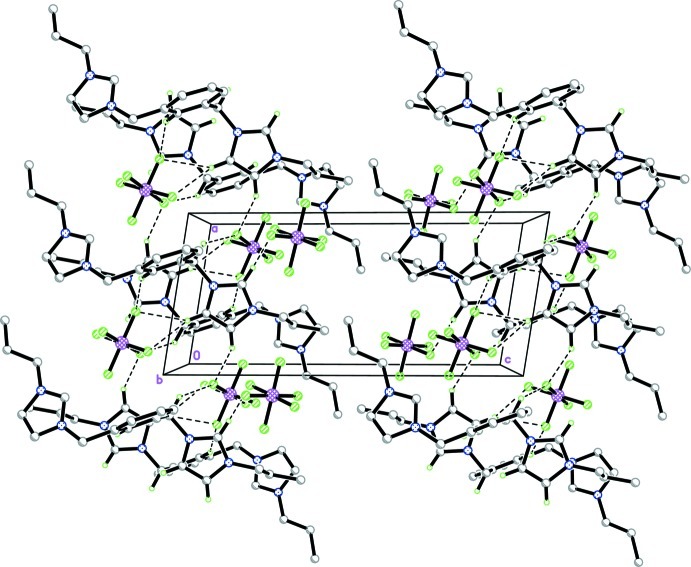
The crystal packing of (I). Dashed lines indicate hydrogen bonds. H atoms not involved in the hydrogen bond interactions have been omitted for clarity.

## supplementary crystallographic information

### Comment

Previously, we have reported crystal structures of *para*-xylyl linked bis-imidazolium salts with propyl (Haque *et al.*, 2011) and benzyl (Haque *et al.*, 2010) substitutions. As part of our studies in this area, we now describe the structure of *meta*-xylyl linked bis-imidazolium salts with propyl substitutions (I).

The assymetric unit unit of (I) is shown in Fig. 1. All parameters in (I) are within normal ranges. The central benzene ring (C5—-C10) makes a dihedral angles of 70.18 (11)° and 69.83 (11)° with the N1—N2/C1—C3 and N3—N4/C12—C14 imidazole rings, respectively, while the two imidazole ring make an dihedral angle of 40.52 (12)° with each other.

In the crystal, weak C—H···F interactions link the molecules into a two-dimensional network parallel to (001) (Fig. 2). A π–π interaction where *Cg*1···*Cg*1^v^ = 3.601 (1) Å is also observed (*Cg*1 = C5—C10, symmetry code: (v) 1 - *x*, 2 - *y*, 2 - *z*).

### Experimental

To a solution of 1,3-bis((1*H*-imidazol-1-yl)methyl)benzene (2.20 g, 0.009 mol) in 30 ml of acetonitrile, 1-bromopropane (2.25 g, 0.018 mol) was added. The mixture was refluxed at 363 K for 24 h. The resultant yellow thick liquid was decanted, washed with fresh acetonitrile (2 *x* 5 ml) and converted directly to its hexafluorophosphate counterpart by metathesis reaction using KPF_6_ (3.31 g, 0.018 mol) in 40 ml of methanol/water. The white precipitates were collected, washed with fresh methanol (2 *x* 3 ml) to give the product as a white solid (4.02 g, 73%). *M*.p 418–420 K. Crystals suitable for X-ray diffraction studies were obtained by slow diffusion method of the salt solution by using diethyl ether and acetonitrile at ambient temperature.

### Refinement

All H atoms attached to C atoms were fixed gemoterically and refined as riding with C—H = 0.95–0.99 Å and *U*_iso_(H) = 1.2*U*_eq_(C) or 1.5*U*_eq_(C) for methyl H atoms. A rotating group model was applied to the methyl group.

### Crystal data

**Table:**

C_20_H_28_N_4_^2+^·2PF_6_^−^	*Z* = 2
*M**_r_* = 614.40	*F*(000) = 628
Triclinic, *P*1	*D*_x_ = 1.572 Mg m^−^^3^
Hall symbol: -P 1	Mo *K*α radiation, λ = 0.71073 Å
*a* = 7.2623 (2) Å	Cell parameters from 5331 reflections
*b* = 11.3926 (3) Å	θ = 2.6–30.0°
*c* = 15.9191 (4) Å	µ = 0.27 mm^−^^1^
α = 86.157 (1)°	*T* = 100 K
β = 80.917 (2)°	Plate, colourless
γ = 88.946 (2)°	0.33 × 0.15 × 0.07 mm
*V* = 1297.61 (6) Å^3^	

### Data collection

**Table:**

Bruker SMART APEXII CCD area-detector diffractometer	7499 independent reflections
Radiation source: fine-focus sealed tube	5364 reflections with *I* > 2σ(*I*)
Graphite monochromator	*R*_int_ = 0.037
φ and ω scans	θ_max_ = 30.0°, θ_min_ = 2.1°
Absorption correction: multi-scan (*SADABS*; Bruker, 2009)	*h* = −10→9
*T*_min_ = 0.916, *T*_max_ = 0.980	*k* = −16→16
20758 measured reflections	*l* = −22→22

### Refinement

**Table:**

Refinement on *F*^2^	Primary atom site location: structure-invariant direct methods
Least-squares matrix: full	Secondary atom site location: difference Fourier map
*R*[*F*^2^ > 2σ(*F*^2^)] = 0.051	Hydrogen site location: inferred from neighbouring sites
*wR*(*F*^2^) = 0.126	H-atom parameters constrained
*S* = 1.04	*w* = 1/[σ^2^(*F*_o_^2^) + (0.0483*P*)^2^ + 0.7762*P*] where *P* = (*F*_o_^2^ + 2*F*_c_^2^)/3
7499 reflections	(Δ/σ)_max_ = 0.001
345 parameters	Δρ_max_ = 0.60 e Å^−^^3^
0 restraints	Δρ_min_ = −0.47 e Å^−^^3^

### Special details

**Table:**

Experimental. The crystal was placed in the cold stream of an Oxford Cryosystems Cobra open-flow nitrogen cryostat (Cosier & Glazer, 1986) operating at 100.0 (1) K.
Geometry. All e.s.d.'s (except the e.s.d. in the dihedral angle between two l.s. planes) are estimated using the full covariance matrix. The cell e.s.d.'s are taken into account individually in the estimation of e.s.d.'s in distances, angles and torsion angles; correlations between e.s.d.'s in cell parameters are only used when they are defined by crystal symmetry. An approximate (isotropic) treatment of cell e.s.d.'s is used for estimating e.s.d.'s involving l.s. planes.
Refinement. Refinement of *F*^2^ against ALL reflections. The weighted *R*-factor *wR* and goodness of fit *S* are based on *F*^2^, conventional *R*-factors *R* are based on *F*, with *F* set to zero for negative *F*^2^. The threshold expression of *F*^2^ > σ(*F*^2^) is used only for calculating *R*-factors(gt) *etc*. and is not relevant to the choice of reflections for refinement. *R*-factors based on *F*^2^ are statistically about twice as large as those based on *F*, and *R*- factors based on ALL data will be even larger.

### Fractional atomic coordinates and isotropic or equivalent isotropic displacement parameters (Å^2^)

**Table:**

	*x*	*y*	*z*	*U*_iso_*/*U*_eq_	
P1	0.85664 (7)	0.82097 (5)	0.31016 (3)	0.02059 (12)	
F1	0.7969 (2)	0.91353 (17)	0.38048 (10)	0.0561 (5)	
F2	0.8446 (3)	0.71820 (17)	0.38131 (13)	0.0755 (7)	
F3	0.9150 (2)	0.73192 (17)	0.23815 (12)	0.0654 (6)	
F4	0.8665 (2)	0.92672 (15)	0.23893 (10)	0.0520 (5)	
F5	0.64195 (17)	0.80311 (12)	0.30128 (8)	0.0300 (3)	
F6	1.07061 (17)	0.84028 (12)	0.31794 (9)	0.0319 (3)	
P2	0.81889 (7)	0.34677 (4)	0.16519 (3)	0.01901 (12)	
F7	0.7426 (2)	0.22554 (12)	0.21327 (9)	0.0381 (3)	
F8	0.7654 (2)	0.41275 (13)	0.25124 (8)	0.0371 (3)	
F9	0.89514 (18)	0.46793 (11)	0.11586 (9)	0.0320 (3)	
F10	0.87151 (19)	0.28187 (12)	0.07852 (8)	0.0324 (3)	
F11	0.61511 (16)	0.37691 (11)	0.14134 (8)	0.0248 (3)	
F12	1.02204 (18)	0.31796 (11)	0.18866 (8)	0.0299 (3)	
N1	0.3946 (2)	0.60725 (14)	1.20589 (10)	0.0192 (3)	
N2	0.5531 (2)	0.64301 (14)	1.08018 (10)	0.0189 (3)	
N3	0.6765 (2)	0.94445 (15)	0.72832 (10)	0.0217 (4)	
N4	0.8876 (2)	0.85736 (15)	0.64240 (11)	0.0223 (4)	
C1	0.5623 (3)	0.63404 (16)	1.16302 (12)	0.0184 (4)	
H1A	0.6712	0.6450	1.1876	0.022*	
C2	0.2740 (3)	0.59791 (18)	1.14801 (13)	0.0238 (4)	
H2A	0.1454	0.5789	1.1609	0.029*	
C3	0.3726 (3)	0.62089 (18)	1.06960 (13)	0.0242 (4)	
H3A	0.3262	0.6217	1.0170	0.029*	
C4	0.7093 (3)	0.67501 (18)	1.01161 (13)	0.0243 (4)	
H4A	0.7001	0.6296	0.9615	0.029*	
H4B	0.8286	0.6535	1.0315	0.029*	
C5	0.7088 (3)	0.80473 (17)	0.98537 (12)	0.0178 (4)	
C6	0.7713 (3)	0.88610 (19)	1.03618 (13)	0.0230 (4)	
H6A	0.8116	0.8599	1.0883	0.028*	
C7	0.7746 (3)	1.00469 (19)	1.01086 (13)	0.0245 (4)	
H7A	0.8155	1.0600	1.0459	0.029*	
C8	0.7182 (3)	1.04277 (17)	0.93422 (13)	0.0233 (4)	
H8A	0.7241	1.1240	0.9162	0.028*	
C9	0.6531 (3)	0.96306 (17)	0.88368 (12)	0.0199 (4)	
C10	0.6477 (3)	0.84405 (17)	0.91006 (12)	0.0187 (4)	
H10A	0.6018	0.7892	0.8762	0.022*	
C11	0.5836 (3)	1.0054 (2)	0.80269 (13)	0.0277 (5)	
H11A	0.4475	0.9925	0.8095	0.033*	
H11B	0.6056	1.0911	0.7922	0.033*	
C12	0.6042 (3)	0.9320 (2)	0.65459 (13)	0.0274 (5)	
H12A	0.4841	0.9567	0.6437	0.033*	
C13	0.7359 (3)	0.8779 (2)	0.60076 (14)	0.0272 (5)	
H13A	0.7261	0.8577	0.5447	0.033*	
C14	0.8479 (3)	0.89906 (18)	0.71901 (12)	0.0224 (4)	
H14A	0.9288	0.8969	0.7605	0.027*	
C15	0.3498 (3)	0.58921 (18)	1.29920 (12)	0.0249 (4)	
H15A	0.2461	0.6427	1.3200	0.030*	
H15B	0.4596	0.6102	1.3247	0.030*	
C16	0.2949 (4)	0.4642 (2)	1.32888 (14)	0.0335 (5)	
H16A	0.3968	0.4096	1.3077	0.040*	
H16B	0.1820	0.4432	1.3057	0.040*	
C17	0.2563 (5)	0.4522 (2)	1.42580 (15)	0.0471 (7)	
H17A	0.2182	0.3715	1.4448	0.071*	
H17B	0.1562	0.5069	1.4465	0.071*	
H17C	0.3695	0.4706	1.4485	0.071*	
C18	1.0638 (3)	0.7974 (2)	0.61052 (14)	0.0301 (5)	
H18A	1.0414	0.7117	0.6128	0.036*	
H18B	1.1555	0.8108	0.6487	0.036*	
C19	1.1443 (4)	0.8377 (2)	0.52238 (15)	0.0411 (6)	
H19A	1.1590	0.9242	0.5189	0.049*	
H19B	1.0570	0.8190	0.4834	0.049*	
C20	1.3308 (3)	0.7813 (3)	0.49349 (16)	0.0404 (6)	
H20A	1.3743	0.8062	0.4336	0.061*	
H20B	1.3184	0.6955	0.4994	0.061*	
H20C	1.4210	0.8054	0.5286	0.061*	

### Atomic displacement parameters (Å^2^)

**Table:**

	*U*^11^	*U*^22^	*U*^33^	*U*^12^	*U*^13^	*U*^23^
P1	0.0202 (3)	0.0195 (3)	0.0224 (2)	−0.0027 (2)	−0.00378 (19)	−0.00144 (19)
F1	0.0388 (9)	0.0833 (13)	0.0534 (10)	0.0118 (9)	−0.0134 (7)	−0.0463 (9)
F2	0.0605 (12)	0.0671 (12)	0.1003 (15)	−0.0310 (10)	−0.0419 (11)	0.0612 (11)
F3	0.0311 (9)	0.0775 (13)	0.0976 (14)	0.0128 (8)	−0.0164 (9)	−0.0670 (11)
F4	0.0490 (10)	0.0548 (10)	0.0547 (10)	−0.0246 (8)	−0.0270 (8)	0.0303 (8)
F5	0.0203 (6)	0.0327 (7)	0.0374 (7)	−0.0052 (5)	−0.0051 (5)	−0.0030 (6)
F6	0.0229 (7)	0.0327 (7)	0.0425 (7)	−0.0033 (6)	−0.0115 (5)	−0.0046 (6)
P2	0.0217 (3)	0.0146 (2)	0.0211 (2)	−0.00125 (19)	−0.00516 (19)	−0.00024 (18)
F7	0.0439 (9)	0.0262 (7)	0.0438 (8)	−0.0106 (6)	−0.0115 (6)	0.0153 (6)
F8	0.0394 (8)	0.0464 (9)	0.0287 (7)	0.0076 (7)	−0.0103 (6)	−0.0165 (6)
F9	0.0267 (7)	0.0213 (6)	0.0476 (8)	−0.0056 (5)	−0.0100 (6)	0.0115 (6)
F10	0.0344 (7)	0.0357 (8)	0.0292 (7)	0.0097 (6)	−0.0080 (5)	−0.0126 (6)
F11	0.0209 (6)	0.0239 (6)	0.0311 (6)	−0.0015 (5)	−0.0071 (5)	−0.0041 (5)
F12	0.0274 (7)	0.0274 (7)	0.0376 (7)	0.0037 (5)	−0.0147 (5)	−0.0006 (5)
N1	0.0229 (9)	0.0153 (8)	0.0190 (8)	−0.0028 (7)	−0.0024 (6)	−0.0009 (6)
N2	0.0243 (9)	0.0137 (7)	0.0181 (7)	0.0001 (6)	−0.0023 (6)	0.0007 (6)
N3	0.0218 (9)	0.0200 (8)	0.0220 (8)	0.0009 (7)	−0.0010 (6)	0.0031 (6)
N4	0.0200 (9)	0.0221 (9)	0.0230 (8)	0.0010 (7)	0.0001 (6)	0.0038 (7)
C1	0.0206 (10)	0.0156 (9)	0.0192 (9)	−0.0025 (7)	−0.0031 (7)	−0.0008 (7)
C2	0.0214 (10)	0.0200 (10)	0.0311 (10)	−0.0023 (8)	−0.0077 (8)	−0.0013 (8)
C3	0.0281 (11)	0.0202 (10)	0.0267 (10)	0.0014 (8)	−0.0114 (8)	−0.0023 (8)
C4	0.0310 (12)	0.0180 (10)	0.0207 (9)	0.0036 (8)	0.0045 (8)	0.0020 (7)
C5	0.0182 (9)	0.0151 (9)	0.0187 (8)	0.0012 (7)	0.0013 (7)	−0.0012 (7)
C6	0.0177 (10)	0.0292 (11)	0.0221 (9)	0.0033 (8)	−0.0023 (7)	−0.0055 (8)
C7	0.0182 (10)	0.0232 (10)	0.0321 (11)	−0.0033 (8)	0.0003 (8)	−0.0108 (8)
C8	0.0200 (10)	0.0142 (9)	0.0324 (11)	−0.0032 (8)	0.0065 (8)	−0.0025 (8)
C9	0.0189 (10)	0.0183 (9)	0.0204 (9)	0.0020 (8)	0.0019 (7)	0.0001 (7)
C10	0.0210 (10)	0.0149 (9)	0.0194 (9)	−0.0019 (7)	0.0008 (7)	−0.0032 (7)
C11	0.0309 (12)	0.0260 (11)	0.0234 (10)	0.0098 (9)	0.0008 (8)	0.0040 (8)
C12	0.0218 (11)	0.0329 (12)	0.0277 (10)	−0.0001 (9)	−0.0056 (8)	0.0014 (9)
C13	0.0247 (11)	0.0310 (12)	0.0259 (10)	−0.0023 (9)	−0.0046 (8)	−0.0004 (9)
C14	0.0232 (10)	0.0203 (10)	0.0220 (9)	−0.0013 (8)	−0.0013 (8)	0.0047 (7)
C15	0.0329 (12)	0.0213 (10)	0.0185 (9)	−0.0059 (9)	0.0024 (8)	−0.0010 (7)
C16	0.0472 (15)	0.0238 (11)	0.0272 (11)	−0.0086 (10)	0.0006 (10)	0.0018 (9)
C17	0.075 (2)	0.0334 (14)	0.0272 (12)	−0.0120 (14)	0.0062 (12)	0.0060 (10)
C18	0.0268 (12)	0.0320 (12)	0.0282 (11)	0.0085 (9)	0.0027 (9)	0.0029 (9)
C19	0.0433 (15)	0.0446 (15)	0.0290 (12)	0.0090 (12)	0.0103 (10)	0.0050 (11)
C20	0.0320 (13)	0.0547 (17)	0.0333 (12)	−0.0028 (12)	0.0037 (10)	−0.0140 (12)

### Geometric parameters (Å, º)

**Table:**

P1—F2	1.5672 (16)	C6—C7	1.383 (3)
P1—F3	1.5842 (15)	C6—H6A	0.9500
P1—F4	1.5926 (15)	C7—C8	1.388 (3)
P1—F1	1.5942 (15)	C7—H7A	0.9500
P1—F6	1.5985 (13)	C8—C9	1.389 (3)
P1—F5	1.6054 (13)	C8—H8A	0.9500
P2—F8	1.5973 (13)	C9—C10	1.392 (3)
P2—F7	1.5976 (13)	C9—C11	1.504 (3)
P2—F10	1.5988 (13)	C10—H10A	0.9500
P2—F12	1.6012 (13)	C11—H11A	0.9900
P2—F9	1.6044 (13)	C11—H11B	0.9900
P2—F11	1.6104 (13)	C12—C13	1.348 (3)
N1—C1	1.329 (2)	C12—H12A	0.9500
N1—C2	1.378 (3)	C13—H13A	0.9500
N1—C15	1.471 (2)	C14—H14A	0.9500
N2—C1	1.328 (2)	C15—C16	1.511 (3)
N2—C3	1.378 (3)	C15—H15A	0.9900
N2—C4	1.477 (2)	C15—H15B	0.9900
N3—C14	1.329 (3)	C16—C17	1.521 (3)
N3—C12	1.376 (3)	C16—H16A	0.9900
N3—C11	1.477 (3)	C16—H16B	0.9900
N4—C14	1.326 (3)	C17—H17A	0.9800
N4—C13	1.382 (3)	C17—H17B	0.9800
N4—C18	1.473 (3)	C17—H17C	0.9800
C1—H1A	0.9500	C18—C19	1.478 (3)
C2—C3	1.349 (3)	C18—H18A	0.9900
C2—H2A	0.9500	C18—H18B	0.9900
C3—H3A	0.9500	C19—C20	1.507 (4)
C4—C5	1.509 (3)	C19—H19A	0.9900
C4—H4A	0.9900	C19—H19B	0.9900
C4—H4B	0.9900	C20—H20A	0.9800
C5—C10	1.387 (3)	C20—H20B	0.9800
C5—C6	1.397 (3)	C20—H20C	0.9800
			
F2—P1—F3	91.17 (13)	C6—C7—C8	119.91 (18)
F2—P1—F4	179.03 (12)	C6—C7—H7A	120.0
F3—P1—F4	89.73 (11)	C8—C7—H7A	120.0
F2—P1—F1	90.50 (12)	C7—C8—C9	120.51 (19)
F3—P1—F1	178.29 (11)	C7—C8—H8A	119.7
F4—P1—F1	88.60 (10)	C9—C8—H8A	119.7
F2—P1—F6	90.08 (8)	C8—C9—C10	119.28 (18)
F3—P1—F6	90.72 (8)	C8—C9—C11	120.31 (19)
F4—P1—F6	90.28 (8)	C10—C9—C11	120.39 (18)
F1—P1—F6	89.63 (8)	C5—C10—C9	120.63 (17)
F2—P1—F5	90.75 (8)	C5—C10—H10A	119.7
F3—P1—F5	89.25 (8)	C9—C10—H10A	119.7
F4—P1—F5	88.89 (8)	N3—C11—C9	112.25 (17)
F1—P1—F5	90.37 (8)	N3—C11—H11A	109.2
F6—P1—F5	179.16 (8)	C9—C11—H11A	109.2
F8—P2—F7	90.62 (8)	N3—C11—H11B	109.2
F8—P2—F10	179.39 (8)	C9—C11—H11B	109.2
F7—P2—F10	89.82 (8)	H11A—C11—H11B	107.9
F8—P2—F12	90.01 (7)	C13—C12—N3	107.08 (19)
F7—P2—F12	90.40 (7)	C13—C12—H12A	126.5
F10—P2—F12	90.41 (7)	N3—C12—H12A	126.5
F8—P2—F9	90.01 (8)	C12—C13—N4	107.17 (19)
F7—P2—F9	179.32 (8)	C12—C13—H13A	126.4
F10—P2—F9	89.55 (8)	N4—C13—H13A	126.4
F12—P2—F9	89.86 (7)	N4—C14—N3	108.95 (18)
F8—P2—F11	89.88 (7)	N4—C14—H14A	125.5
F7—P2—F11	90.08 (7)	N3—C14—H14A	125.5
F10—P2—F11	89.70 (7)	N1—C15—C16	113.01 (17)
F12—P2—F11	179.50 (8)	N1—C15—H15A	109.0
F9—P2—F11	89.65 (7)	C16—C15—H15A	109.0
C1—N1—C2	108.30 (16)	N1—C15—H15B	109.0
C1—N1—C15	124.94 (17)	C16—C15—H15B	109.0
C2—N1—C15	126.75 (17)	H15A—C15—H15B	107.8
C1—N2—C3	108.44 (16)	C15—C16—C17	109.66 (19)
C1—N2—C4	125.44 (17)	C15—C16—H16A	109.7
C3—N2—C4	126.10 (17)	C17—C16—H16A	109.7
C14—N3—C12	108.51 (17)	C15—C16—H16B	109.7
C14—N3—C11	125.94 (18)	C17—C16—H16B	109.7
C12—N3—C11	125.42 (18)	H16A—C16—H16B	108.2
C14—N4—C13	108.28 (18)	C16—C17—H17A	109.5
C14—N4—C18	124.28 (18)	C16—C17—H17B	109.5
C13—N4—C18	127.43 (18)	H17A—C17—H17B	109.5
N2—C1—N1	108.94 (17)	C16—C17—H17C	109.5
N2—C1—H1A	125.5	H17A—C17—H17C	109.5
N1—C1—H1A	125.5	H17B—C17—H17C	109.5
C3—C2—N1	107.23 (18)	N4—C18—C19	113.54 (19)
C3—C2—H2A	126.4	N4—C18—H18A	108.9
N1—C2—H2A	126.4	C19—C18—H18A	108.9
C2—C3—N2	107.09 (17)	N4—C18—H18B	108.9
C2—C3—H3A	126.5	C19—C18—H18B	108.9
N2—C3—H3A	126.5	H18A—C18—H18B	107.7
N2—C4—C5	111.85 (16)	C18—C19—C20	112.5 (2)
N2—C4—H4A	109.2	C18—C19—H19A	109.1
C5—C4—H4A	109.2	C20—C19—H19A	109.1
N2—C4—H4B	109.2	C18—C19—H19B	109.1
C5—C4—H4B	109.2	C20—C19—H19B	109.1
H4A—C4—H4B	107.9	H19A—C19—H19B	107.8
C10—C5—C6	119.44 (18)	C19—C20—H20A	109.5
C10—C5—C4	120.15 (17)	C19—C20—H20B	109.5
C6—C5—C4	120.41 (18)	H20A—C20—H20B	109.5
C7—C6—C5	120.19 (19)	C19—C20—H20C	109.5
C7—C6—H6A	119.9	H20A—C20—H20C	109.5
C5—C6—H6A	119.9	H20B—C20—H20C	109.5
			
C3—N2—C1—N1	−0.1 (2)	C8—C9—C10—C5	−0.9 (3)
C4—N2—C1—N1	178.13 (17)	C11—C9—C10—C5	−178.95 (18)
C2—N1—C1—N2	0.5 (2)	C14—N3—C11—C9	−28.5 (3)
C15—N1—C1—N2	179.56 (17)	C12—N3—C11—C9	156.03 (19)
C1—N1—C2—C3	−0.6 (2)	C8—C9—C11—N3	126.7 (2)
C15—N1—C2—C3	−179.71 (19)	C10—C9—C11—N3	−55.2 (3)
N1—C2—C3—N2	0.5 (2)	C14—N3—C12—C13	0.1 (2)
C1—N2—C3—C2	−0.3 (2)	C11—N3—C12—C13	176.22 (19)
C4—N2—C3—C2	−178.50 (18)	N3—C12—C13—N4	0.3 (2)
C1—N2—C4—C5	−95.3 (2)	C14—N4—C13—C12	−0.5 (2)
C3—N2—C4—C5	82.7 (2)	C18—N4—C13—C12	178.2 (2)
N2—C4—C5—C10	−103.8 (2)	C13—N4—C14—N3	0.5 (2)
N2—C4—C5—C6	76.8 (2)	C18—N4—C14—N3	−178.18 (18)
C10—C5—C6—C7	−0.9 (3)	C12—N3—C14—N4	−0.4 (2)
C4—C5—C6—C7	178.52 (18)	C11—N3—C14—N4	−176.51 (18)
C5—C6—C7—C8	−0.9 (3)	C1—N1—C15—C16	−114.1 (2)
C6—C7—C8—C9	1.9 (3)	C2—N1—C15—C16	64.8 (3)
C7—C8—C9—C10	−1.0 (3)	N1—C15—C16—C17	178.4 (2)
C7—C8—C9—C11	177.10 (19)	C14—N4—C18—C19	−135.0 (2)
C6—C5—C10—C9	1.8 (3)	C13—N4—C18—C19	46.6 (3)
C4—C5—C10—C9	−177.64 (18)	N4—C18—C19—C20	176.0 (2)

### Hydrogen-bond geometry (Å, º)

**Table:**

*D*—H···*A*	*D*—H	H···*A*	*D*···*A*	*D*—H···*A*
C1—H1*A*···F3^i^	0.95	2.32	3.238 (3)	162
C2—H2*A*···F9^ii^	0.95	2.46	3.274 (3)	144
C3—H3*A*···F10^iii^	0.95	2.45	3.291 (2)	148
C3—H3*A*···F11^iii^	0.95	2.49	3.344 (2)	150
C4—H4*B*···F9^i^	0.99	2.51	3.195 (2)	126
C10—H10*A*···F11^iii^	0.95	2.54	3.405 (2)	151
C18—H18*B*···F7^iv^	0.99	2.43	3.324 (3)	149

Symmetry codes: (i) *x*, *y*, *z*+1; (ii) *x*−1, *y*, *z*+1; (iii) −*x*+1, −*y*+1, −*z*+1; (iv) −*x*+2, −*y*+1, −*z*+1.
